# Patient-, implant- and prosthetic-related factors on peri-implant mucositis and bone loss

**DOI:** 10.1590/1807-3107bor-2024.vol38.0040

**Published:** 2024-05-13

**Authors:** Lélis Gustavo NÍCOLI, Carolina Mendonça de Almeida MALZONI, Paulo Fermino da COSTA NETO, Claudio MARCANTONIO, Suzane Cristina PIGOSSI, Cassiano Kuchenbecker RÖSING, Francisco Wilker Mustafa Gomes MUNIZ, Marcelo GONÇALVES, Daniela Leal ZANDIM-BARCELOS, Elcio MARCANTONIO JUNIOR

**Affiliations:** (a) Universidade Estadual Paulista – Unesp, School of Dentistry at Araraquara, Department of Diagnosis and Surgery, Araraquara, SP, Brazil.; (b) Universidade Estadual Paulista – Unesp, School of Dentistry at Araraquara, Department of Restorative Dentistry, Universidade Estadual Paulista - UNESP, Araraquara, SP, Brazil.; (c) Universidade de Araraquara – Uniara, Dentistry Graduate Program, Araraquara, SP, Brazil.; (d) Federal University of Uberlandia - UFU, School of Dentistry, Department of Periodontology and Implantodontoly, Uberlandia, MG, Brazil; (e) Universidade Federal do Rio Grande do Sul – UFRGS, School of Dentistry, Department of Conservative Dentistry, Porto Alegre, RS, Brazil.; (f) Universidade Federal de Pelotas – UFPel, School of Dentisty,Department of Semiology and Clinics, Pelotas, RS, Brazil.

**Keywords:** Dental Implants, Risk Factors, Mucositis

## Abstract

Peri-implant diseases, including peri-implant mucositis (PIM) and peri-implantitis, are a chronic inflammatory disorder triggered by bacterial biofilm in susceptible hosts. Potential risk factors for peri-implant diseases include smoking, dental plaque accumulation, poor oral hygiene, genetics, and absence of peri-implant keratinized mucosa. This cohort study aimed to evaluate the influence of patient-, implant-, and prosthetic-related factors on PIM and peri-implant bone loss (PBL) around dental implants after 1 year of loading. A total of 54 subjects (22 males and 32 females) were included in the study. Peri-implant clinical parameters were assessed and standardized periapical radiographs of each dental implant were obtained 15 days after the definitive prosthesis installation (baseline) and at 3, 6, and 12 months of follow-up. A total of 173 implants were evaluated. PIM affected 44.8% of the implants and no significant association was found between the investigated parameters and PIM incidence, except for type of implant connection. A significantly higher incidence of PIM (80.0%) was observed for implants with internal hexagon connection type after 1 year of follow-up (p = 0.015). Moreover, a mean PBL of 0.35 ± 1.89 mm was observed and no dental implant was affected by peri-implantitis after 1 year of function. No specific influence of patient, implant, or prosthetic factors on PBL was observed. No association was found between the occurrence of PIM/PBL and the patient-, implant-, and prosthetic-related factors investigated in this cohort study, except for the type of dental-implant connection.

## Introduction

Over the last few decades, dental implant restorations have achieved high long-term success rates and elevated the standards for rehabilitation of edentulous patients.^1^ However, marginal bone loss around dental implants may pose a risk to implant longevity, once it can result in complications such as implant fractures, soft tissue recession, and, ultimately, implant loss.^2^

In the classic implant success criteria, the threshold for acceptable bone loss is dynamic, allowing less than 1.5 mm of bone loss during the first year of loading and less than 0.2 mm annually after the first year.^3^ It is well documented that this initial remodeling of the implant marginal bone occurs after functional loading to create a biological width between the dental implant platform and the bone crest.^4^ However, the presence of bacteria on the implant surface could also result in an inflammatory response and bone resorption.^2,5^

Peri-implant mucositis is a reversible inflammatory condition that causes redness and swelling of the soft tissue around dental implants without clinical evidence of bone loss.^6^ If left untreated, peri-implant mucositis may advance to peri-implantitis. The term peri-implantitis was suggested to describe a destructive infectious pathology around dental implants that results in bone loss.^2,7^ and inflammatory conditions with bleeding on probing observed after the physiological remodeling period.^8^ Peri-implantitis is defined as a plaque-induced and host-mediated damaging process that is affected by modifiable and non-modifiable local, systemic, and environmental factors.^9^

Marginal bone loss and peri-implant diseases have been shown to be affected by a diversity of implant-related properties, patient-related factors, and prosthetic characteristics.^10^ Regarding implant and prosthetic characteristics, implant position and angulation, implant surface design, and prosthesis design have been related to marginal bone loss.^11,12^ Patient-related factors comprising dental plaque accumulation, poor oral hygiene, history of previous periodontal disease, diabetes, alcohol consumption, smoking, genetics, and absence of peri-implant keratinized mucosa (KM) have also been associated with peri-implant diseases.^13^ Identification of these risk indicators is necessary to prevent pathologic bone loss, once individuals who present numerous bone loss risk indicators should be supervised more frequently to prevent disease progression.^2^

Therefore, this cohort study aimed to determine the occurrence of peri-implant mucositis and peri-implant bone loss in implants after 1 year of function, as well as the influence of patient-, implant-, and prosthetic-related factors on peri-implant mucositis and peri-implant bone loss.

## Methodology

### Study sample

The present cohort study described the data from partially or fully edentulous patients who were rehabilitated with implant supported-prosthesis. The patients were recruited from 2014 to 2016 at the Implant Dentistry clinic of the School of Dentistry at Araraquara. All participants were informed about the importance of supportive post-implant therapy for the long-term success of their dental implant rehabilitation treatment. This study was approved by the Human Research Ethics Committee of the School of Dentistry in Araraquara (CAAE #41357514.5.0000.5416) and was performed following the principles stated in the Declaration of Helsinki. All patients were informed about the objectives of the study and spontaneously agreed to participate by signing the free and informed consent form.

### Clinical examination

On the first visit, before the clinical examination, demographic data (age, gender), systemic/behavioral data, and characteristics of the implants installed, prosthetic rehabilitation, abutments, and radiographic adaptation were collected. All these data were registered in a record chart specifically designed for this study.

The following periodontal and peri-implant clinical parameters were documented 15 days after the definitive prosthesis installation (baseline) and at the follow-up visits (3, 6, and 12 months): presence or absence of visible plaque, presence or absence of marginal bleeding, probing depth (PD), presence or absence of bleeding on probing (BOP), and suppuration. These parameters were evaluated at six sites around the tooth or implant (mesiobuccal, mid-buccal, distobuccal, mesiolingual/palatal, mid-lingual/palatal, and distolingual/palatal), with exception of visible plaque and marginal bleeding that were verified only at four sites (mesial, buccal, lingual, and distal).

The clinical examination was executed by one calibrated (Wilcoxon test p>0.05; Spearman correlation r = 0.81) examiner (LGN) using a periodontal probe (UNC 15; Hu-Friedy, Chicago, USA). The width of the keratinized mucosa (KM) and gingival thickness at the mid-buccal site of each implant were registered. For the identification of the mucogingival junction line, variances in texture, mobility, and color between the KM and the oral mucosa were examined. After that, the implants were classified as: Score 0 – absence of KM, Score 1 – KM width > 0 mm and ≤ 1 mm, Score 2 – KM width > 1 mm and ≤ 2 mm, or Score 3 – KM width > 2 mm.^14^ Gingival thickness was assessed based on the transparency of the gingival margin using a periodontal probe. If the outline of the underlying periodontal probe could be seen through the gingiva, it was categorized as thin (score: 0); otherwise, it was categorized as thick (score: 1).^15^

The new classification of periodontitis^16^ defines the disease in the presence of interproximal clinical attachment loss (CAL) ≥ 2 mm in non-adjacent teeth or in the presence of buccal/oral CAL ≥3 mm with probing depth ≥3 mm in at least 2 teeth with non-periodontitis-related CAL causes. However, as this study started in 2014, the diagnosis of periodontitis was defined as the presence of four or more teeth with at least one site with PD ≥ 4 mm, BOP, and CAL ≥ 3 mm.^17^

The same condition occurred for peri-implant diseases. The new classification considers peri-implantitis as increased probing depth, associated with BOP, and radiographic bone loss, in addition to conditions observed during the remodeling period.^8^ In this study, peri-implantitis was diagnosed as BOP and/or suppuration in combination with radiographic bone loss ≥2 mm and probing depth ≥5 mm, and peri-implant mucositis was defined as BOP and/or suppuration without bone loss.^18^

### Radiograph exam

Direct digital periapical radiographs were taken for each implant (DigoraUptime, Soredex, Tuusula, Finland) using standardized positioners and the long cone parallel technique 15 days after the definitive prosthesis installation (baseline) and at 3, 6, and 12 months of function. To standardize the periapical radiographs, condensation silicone was applied to fix the positioner and to reproduce the same position of the radiographic film and x-ray machine on the radiographs taken at every time-point. Marginal bone level was measured in the mesial and distal surfaces of each dental implant using a specific software program (ImageJ - version 1.32j / NIH software - Bethesda, USA).^19,20^ The measure was made from implant platform to the most critical level of bone loss. The mean of the mesial and distal measurements corresponded to the marginal bone level in each implant.^21^ The values of peri-implant bone level were assumed as positive when the marginal bone was coronal to the implant platform and negative when the marginal bone was positioned apical to the platform. To compensate image distortions, a linear calibration of the software was made for each implant based on their actual size.^22^ The marginal bone level measurements were executed by two trained and calibrated evaluators (LGN and PF). The intra- and inter-examiner calibrations were done using the Pearson test. The result was 0.81 for inter-examiner correlation. For the intra-examiner analysis, the correlation was 0.97 for the first evaluator and 0.91 for the second evaluator.

### Statistical analysis

For the analysis of peri-implant mucositis (PIM) occurrence, the total sample was divided into peri-implant health and peri-implant mucositis. The independent age variable was divided into two groups, one with individuals up to 59 years old and the other with individuals at least 60 years old. Thus, the chi-square test was applied to all categorical variables. A value of p < 0.05 was used to determine statistical significance.

For peri-implant bone loss (PBL) evaluation, a mean bone loss of 0.35 ± 1.89 mm, with a median of 0.19 mm, was observed. Therefore, the sample was dichotomized into two groups, one with more than 0.19 mm of peri-implant bone loss and another with less than 0.19 mm of PBL, and associations between PBL and the independent variables was verified by the chi-square test. Univariate analyses of linear regression were performed with all independent variables and PBL. Only those variables with a value of p < 0.20 were included in the multivariate model. Thus, the multivariate model included the following independent variables: gender, type of prosthesis, and width and keratinized mucosa, which remained in the final multivariate model. In all analyses, a value of p <0.05 was used to determine statistical significance. All statistical analyses were performed in SPSS software (SPSS version 18.0, Chicago, USA).

The study power calculation was based on the mean PBL of both groups. An alpha of 5% and mean ± SD bone loss of -0.93 ± 1.24 and 1.60 ± 1.54 in the groups with lower and higher bone loss, respectively. A power of 88.37% for a two-sample mean comparison was determined.

## Results

**Figure 1:**
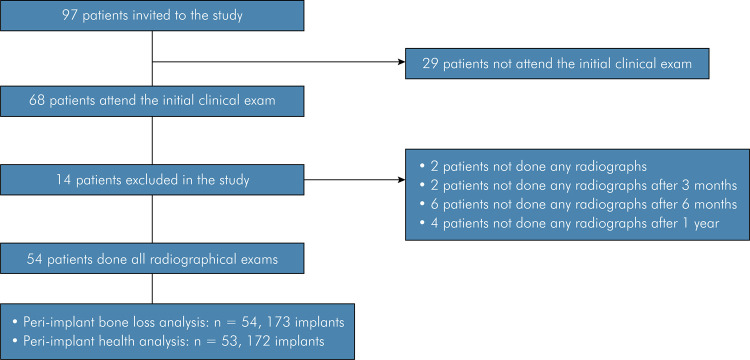
Flow diagram showing included and excluded patients.

A total of 97 patients were recruited for this cohort study, but only 54 subjects (22 men and 32 women), with a mean age of 56.67 ± 8.44 years, were included (Figure 1). A total of 173 implants were evaluated. However, one patient with one implant didn’t return for clinical examinations, only attending the radiological clinic for periapical radiographs. The demographic variables of the patients included in the study are described in Table 1.

**Table 1:** Demographic variables of the patients included in the study.

Demographic variables	
Age (± SD)	56.67 ± 8.44
Gender n (%)	
Female	32 (59.25)
Male	22 (40.74)
Smokers n (%)	4 (7.1)
Ex-smokers n (%)	6 (10.7)
Never smoked n (%)	46 (82.1)
Type 2 diabetes n (%)	3 (5.3)
Cardiovascular disease n (%)	6 (10.7)
Hypothyroidism n (%)	7 (12.5)
History of cancer n (%)	2 (3.5)
Radiotherapy n (%)	1 (1.7%)

The dental implant variables are described in Table 2. Despite similar surface treatment (blasting with acid etching), four Brazilian brands of manufactured implants were used in the sample: 55 implants (31.8%) were from Implacil de Bortoli^®^ (São Paulo, Brazil), 86 implants (49.7%) were from Conexão Sistemas de Próteses^®^ (Arujá, Brazil), 27 (15.6%) were from Neodent^®^ (Curitiba, Brazil), and 5 (2.8%) were from Bionovation^®^ (Bauru, Brazil). Other dental implant variables were also described in Table 2.

**Table 2:** Distribution of treatment variables among the dental implants included in the study.

Dental implant variables	n	%
Total number of implants	173	100
Implacil de Bortoli	55	31.8
Conexão Sistemas de Próteses	86	49.7
Neodent	27	15.6
Bioinnovation^®^	5	2.8
Maxilla	92	53.2
Mandible	81	46.8
External hexagon (EH)	95	54.9
Morse Cone (CM)	63	36.4
Internal hexagon (IH)	15	8.3
Narrow platform ≤ 3.5 mm	41	23.7
Regular platform 3.75 mm, 4 mm e 4.1 mm	131	75.7
Large platform ≥ 4.5 mm	1	5.6
Unitary prostheses	51	29.5
Fixed posterior prosthesis	43	23.9
Fixed anterior prosthesis	31	17.2
Branemark protocol	53	30.6
Cement-retained dental implants	13	7.2
Screw-retained dental implants	165	95.4

**Figure 2:**
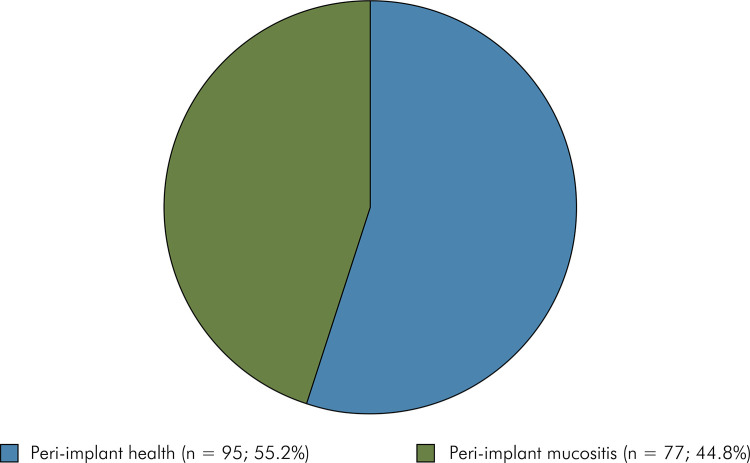
Distribution of implants classified in peri-implant health and peri-implant mucositis after 12 months of follow-up.

**Figure 3:**
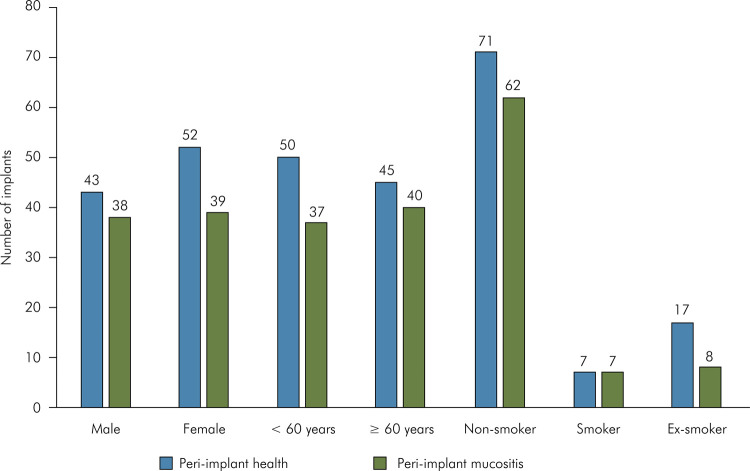
Distribution of implants classified in peri-implant health and peri-implant mucositis based on demographic and behavioral parameters; Chi-square test; Mann-Whitney for age variable.

No case of peri-implantitis was identified after one year of follow-up and 77 implants were diagnosed with mucositis (Figure 2). Thus, to investigate the influence of demographic, behavioral, and clinical parameters on the peri-implant health, the total implant sample was divided into peri-implant health (PIH) [n = 95 (55.2%)] and peri-implant mucositis (PIM) [n = 77 (44.8%)] (Figure 2). No influence of gender, age, and smoking habits was observed on the incidence of peri-implant mucositis (Figure 3).

**Figure 4:**
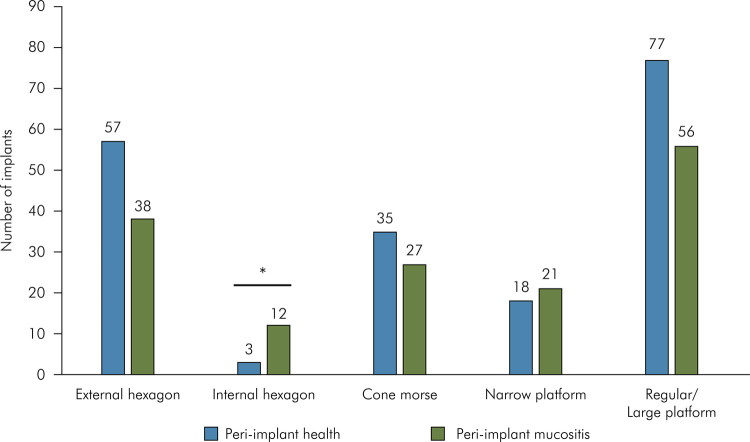
Distribution of implants classified into peri-implant health and peri-implant mucositis based on dental implant characteristics; *p<0.05; Chi-square test.

**Figure 5:**
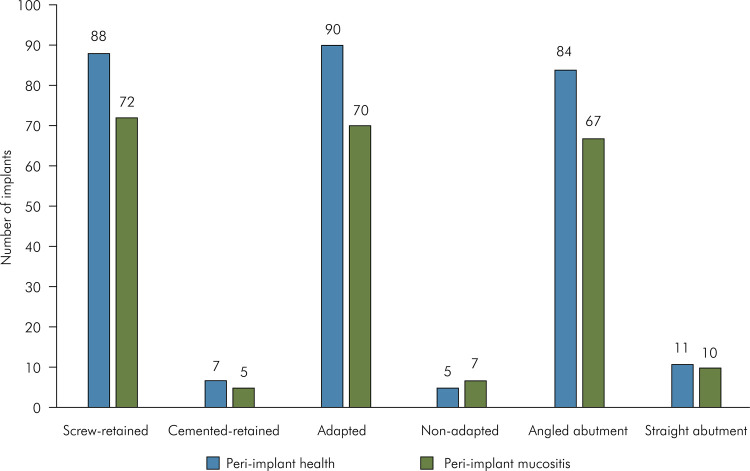
Distribution of implants classified into peri-implant health and peri-implant mucositis based on prosthesis characteristics; Chi-square test.

**Figure 6:**
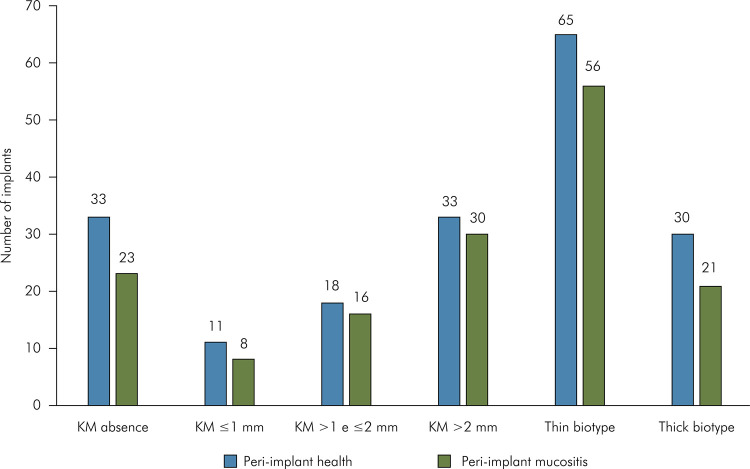
Distribution of implants classified into peri-implant health and peri-implant mucositis based on peri-implant morphology; Chi-square test; KM: peri-implant keratinized mucosa.

Regarding implant characteristics, PIM incidence was significantly higher than PIH in the internal hexagon (IH) connection (p = 0.015). For the other types of implant connection, no differences were observed. Similarly, no differences were found for PIM and PIH based on dental implant platform type (Figure 4), cement- or screw-retained dental implants, abutments type and radiograph adaptation (Figure 5), KM width or gingival biotype (Figure 6), dental arch (mandible versus maxilla) and region (anterior versus posterior) (Table 3), and between the four Brazilian brands of implants included in the study (Table 3).

**Table 3:** Distribution of implants classified into peri-implant health and peri-implant mucositis based on dental arch/region and implants trademarks.

Variable	Peri-implant health	Peri-implant mucositis	p-value
Dental Arch			
Maxillae	54 (56.8)	41 (53.2)	0.637
Mandibule	41 (43.2)	36 (46.8)	
Region			
Anterior	32 (33.7)	24 (31.2)	0.726
Posterior	63 (66.3)	53 (68.8)	
Trademarks			0.306
Implancil	33 (34.7)	22 (28.6)	
Conexão	48 (50.5)	37 (48.1)	
Neodent	13 (13.7)	14 (18.2)	
Bionnovation	1 (1.1)	4 (5.2)	

**Figure 7:**
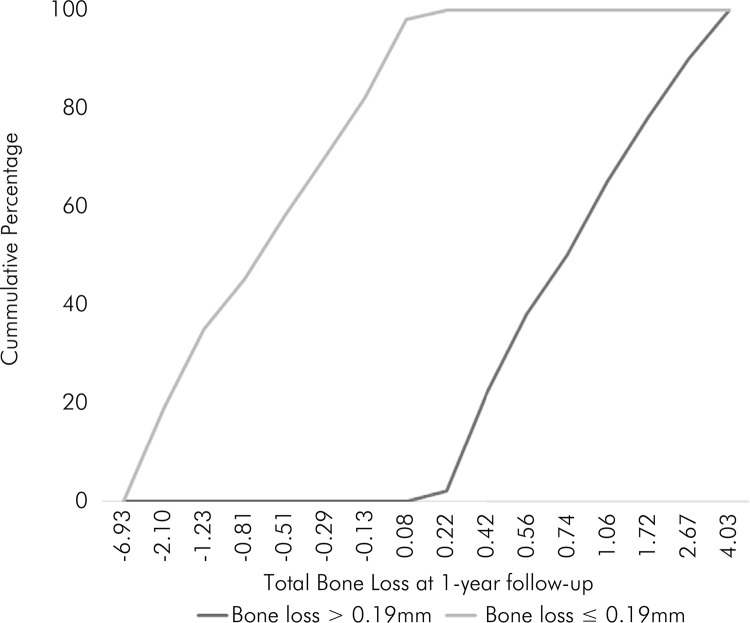
Cumulative percentage of total bone loss in both groups.

No implant loss was observed after 1 year of follow-up. A mean PBL of 0.35 ± 1.89 mm, with a median of 0.19 mm, was observed after one year of loading. To investigate the PBL risk factors the sample was dichotomized into two groups: more than 0.19 mm of peri-implant bone loss (MBL) and less than 0.19 mm of peri-implant bone loss (LBL). Figure 7 shows the cumulative percentage of total bone loss in both groups.

For patient-related factors, no influence of gender, age, and smoking habits on PBL was observed (Table 4). Likewise, for implant-related factors, no difference could be observed between the groups LBL and MBL for the dental implant platform type, prosthesis type, abutment radiographic adaptation, types of dental implant connection, abutment angulation, and implant brand (Table 4). Moreover, no other implant or patient-related factors including KM width, gingival biotype, periodontal condition, and dental region arch interfered with PBL (Table 4). In the univariate analysis, an association was found between KM width, dental arch, and PBL (Table 5). However, this association disappeared in the multi-factor analysis (Table 6).

**Table 4:** Analysis of the association between patient- or implant-related independent variables and peri-implant bone loss.

Variables	Total bone loss ≤ 0.19 mm (n = 86; 49.7%)	Total bone loss > 0.19 mm (n = 87; 50.3%)	p- value

	n (%)	n (%)	
Gender			
Male	43 (52.4)	39 (47.6)	0.496*
Female	43 (47.3)	48 (52.7)	
Age			
Average (minimum, maximum)	56.74 (36.0; 76.0)	57.55 (36.0; 73.0)	0.684#
Categorized age (years)			
< 60	44 (50.6)	43 (49.4)	0.819*
≥ 60	42 (48.8)	44 (51.2)	
Smoking habits			
Non-smoking	68 (50.7)	66 (49.3)	0.825*
Smoker	7 (50.0)	7 (50.0)	
Ex-smoker	11 (44.0)	14 (56.0)	
Implant platform			
Narrow	21 (52.5)	19 (47.5)	0.687*
Regular/Large	65 (48.9)	68 (51.1)	
Prosthesis type			
Screw-retained	79 (49.4)	81 (50.6)	0.757*
Cement-retained	7 (53.8)	6 (46.2)	
Abutment radiographic adaptation			
Adapted	82 (50.9)	79 (49.1)	0.240*
Non-adapted	4 (33.3)	8 (66.7)	
Keratinized mucosa width			
Absence	24 (42.9)	32 (57.1)	0.307*
≤1 mm	11 (57.9)	8 (42.1)	
> 1 and ≤ 2 mm	15 (42.9)	20 (42.1)	
> 2 mm	36 (57.1)	27 (42.9)	
Prosthetic connection			
EH	51 (53.7)	44 (46.3)	0.394*
IH	8 (53.3)	7 (46.7)	
MC	27 (42.9)	36 (57.1)	
Abutment angulation			
Straight	77 (50.7)	75 (49.3)	0.503*
17° ou 30°	9 (42.9)	12 (57.1)	
Gingival biotype			
Thin	59 (48.8)	62 (51.2)	0.703*
Thick	27 (51.9)	25 (48.1)	
Periodontal condition			
Health	39 (54.2)	33 (45.8)	0.577*
Gingivitis and/or Periodontitis	37 (45.7)	44 (54.3)	
Edentulous	10 (50.0)	10 (50.0)	
Dental arch			
Maxillae	51 (59.3)	45 (51.7)	0.316*
Mandible	35 (40.7)	42 (48.3)	
Region			
Anterior	25 (29.1)	32 (36.8)	0.281*
Posterior	61 (70.9)	55 (63.2)	
Trademarks			
Implancil	30 (34.9)	25 (28.7)	0.506*
Conexão	42 (48.8)	44 (50.6)	
Neodent	13 (15.1)	14 (16.1)	
Bionnovation	1 (1.2)	4 (4.6)	

*Chi-square test; #Mann-Whitney test.

**Table 5:** Univariate analysis of total bone loss and patient- or implant-related independent variables.

Variable	Beta (95%IC)	p-value
Gender		
Male	Ref.	
Female	0.014 (-0.019 – 0.046)	0.268
Smoking habits		
Non-smoking	Ref.	
Smoker	-0.124 (-1.181 – 0.933)	0.817
Ex-smoker	0.149 (-0.666 – 0.964)	0.719
Implant Platform		
Narrow	Ref.	
Regular/Large	0.216 (-0.456 – 0.889)	0.526
Prosthesis type		
Screw-retained	Ref.	
Cement-retained	-0.535 (-1.623 – 0.553)	0.333
Abutment radiographic adaptation		
Adapted	Ref.	
Non Adapted	0.259 (-0.857 – 1.376)	0.647
Keratinized mucosa width		
Absence	Ref.	
≤ 1 mm	-0.001 (-0.008 – 0.007)	0.830
> 1 e ≤ 2 mm	-0.004 (-0.011 – 0.002)	0.210
> 2 mm	0.001 (-0.007 – 0.006)	0.828
Prosthetic connection		
EH	Ref.	
IH	-0.187 (-1.127 – 0.754)	0.685
MC	0.001 (-0.632 – 0.634)	0.998
Abutment angulation		
Straight	Ref.	0.981
17° ou 30°	0.011 (-0.859 – 0.880)	
Gingival biotype		
Thin	Ref.	0.274
Thick	-0.343 (-0.960 – 0.274)	
Periodontal condition		
Health		
Gingivitis and/or	Ref.	
Periodontitis	0.253 (-0.362 – 0.868)	0.418
Edentulous	0.366 (-0.566 – 1.298)	0.438
Dental arch		
Maxillae	Ref. 0.706 (0.145 – 1.267)	0.014*
Mandibule		
Position		
Anterior	Ref. 0.210 (-0.393 – 0.813)	0.493
Posterior		
Trademarks		
Implancil	Ref.	
Conexão	-0.001 (-0.011 – 0.008)	0.764
Neodent	0.001 (-0.009 – 0.011)	0.874
Bionnovation	0.001 (-0.008 – 0.011)	0.777

Linear Regression analysis *p < 0.20.

**Table 6:** Multivariate analysis of total bone loss and patient or implant-related independent variables.

Variable	Beta (95%CI)	p-value
Gender		
Male	Ref.	
Female	0.328 (-0.254 – 0.910)	0.268
Prosthesis type		
Screwed	Ref.	
Cemented	-0.535 (-1.623 – 0.553)	0.333
Keratinized mucosa width		
Absence	Ref.	
≤1 mm	-0.001 (-0.008 – 0.007)	0.830
> 1 e ≤2 mm	-0.004 (-0.011 – 0.002)	0.210
> 2 mm	-0.001 (-0.007 – 0.006)	0.828
Dental arch		
Maxillae	Ref.	
Mandible	0.422 (-0.312 – 1.156)	0.258

## Discussion

Because of the importance of identifying the risk factors related to PBL and PIM and prevent complications and ensure long-term success of implant supported-prosthesis, this prospective study analyzed the influence of patient-, implant-, and prosthesis-related factors on PIM and PBL around dental implants after 1 year of loading.

The PIM incidence of 44.8% at the implant level observed in this cohort study corroborates with the results of Meijer et. al.^23^ who reported a PIM incidence of 51.9% in 150 edentulous patients with an implant-retained mandibular overdenture after a 10-year follow-up. Similarly, Lee et al.^24^ conducted a meta-analysis with forty-seven studies and showed a PIM prevalence of 46.83% at the implant level. On the other hand, lower PIM incidences of 20%^22^ and 9.1%^23^ at the implant level have been reported in two prospective 5-year cohort studies including 22^25^ and 60 patients,^26^ respectively. These divergent results could be explained by different case definitions for peri-implant diseases, different population samples, and clinical settings across the studies.

PBL is a requirement in the diagnosis of peri-implantitis, and the stability of the peri-implant bone is regarded an essential factor for implant success.^27^ Mei et al.^26^ evaluated the clinical and radiographic outcomes of rooted, platform-switched, micro-threaded, sandblasted, large-grid, and acid-etched (SLA) surface implants for 5 years. In accordance with our study, no peri-implantitis case was identified and an average marginal bone loss of 0.46±0.27 mm and 0.46 ± 0.32 mm at the mesial and distal aspects, respectively, was detected after 1 year.^26^ After 5 years, the mean marginal bone loss at the mesial aspect was 0.48±0.27 mm and at the distal aspect, it was 0.50 ± 0.35 mm^26^. On the other hand, Fransson et al.^28^ evaluated intra-oral radiographs from 419 implants in 182 patients and reported a mean bone loss of 1.68 mm and a bone loss ≥ 2 mm in 32% of the implants evaluated after one year of function.

The onset and pattern of peri-implantitis have been previously described. Studies have shown that bone loss follow a non-linear pattern and that the bone loss rate increases over time.^28,29^ Derks et al.,^29^after a 9-year follow-up examination of 596 randomly selected individuals with implants, showed a non-linear, accelerating pattern of bone loss at the 105 affected implants. The peri-implantitis onset occurred early, and a total of 70% and 81% of subjects had more than one implant with bone loss >0.5 mm at 2 and 3 years, respectively.^29^ An annual rate of peri-implant bone loss of about 0.4 mm was also observed.^29^ Compared with our study, although no peri-implantitis cases were observed, a similar mean PBL of 0.35 ± 1.89 mm after 1 year of loading was reported. The absence of peri-implantitis cases could be associated with the short follow-up period of our study, once it has been previously suggested that its onset occurs within 3 years of function in the majority of the cases.^29^

No association between demographic and behavioral outcome variables (age, gender, and smoking habits) and PIM and PBL was reported in this study. In contrast, a retrospective study including 101 subjects rehabilitated with dental implants showed a strong association between PIM prevalence and patient age ≥65 years.^30^ This higher PIM occurrence could be due to difficulties in maintaining proper oral hygiene, impaired immunity response, and compromised healing ability in older individuals.^31^

The external hexagon (EH) implants have been the most widely used, but this connection type has some disadvantages, including abutment micromovement, which may result in mechanical and biological complications.^32^ A systematic review with meta-analysis including 11 studies with a total of 1089 implants showed that the internal hexagon (IH) connection implants were associated with lower bone loss than the EH implants.^32^ These results corroborate with a previous study that reported lower values of marginal bone loss in association with IH connection implants.^33^ The internal connections are preferred because of the switching concept, providing lesser micromovements, better stress distributions, and higher survival probability.^34^ On the other hand, studies have shown that the micro-gap of the IH connection is much greater than those for the morse cone (MC) abutment connection.^35^ The less leakage at the implant-abutment interface in MC could explain the lower bone resorption in this system in comparison to the external connection system.^36^ In the present study, the PIM incidence was significantly higher in the IH connection type, but this result must be interpreted with caution, due to the small number of IH connection implants evaluated (n = 15).

In the current study, type of prosthesis, abutment angulation, and absence/presence of prosthesis adaptation had no influence on PIM/PBL. However, previous publications^37,38^ indicated that abutment height, abutment/implant interface, prosthesis contours, retained excess cement, and access for oral hygiene are vital for avoiding PIM and peri-implantitis. The literature demonstrates that cement-retained dental implants have been associated with a higher risk of peri-implantitis.^2,11,12^ This association probably occurs due to residual cement that acts as a contributing factor for late PBL. The rough surface of the cement favors the accumulation of microorganism resulting in tissue inflammation and bone loss.^12^ A systematic review and meta-analysis including nine studies evaluated and compared peri-implant bone loss in cement- and screw-retained dental implants.^11^ A mean marginal bone loss of 0.53 mm (0.31–0.76 mm) was reported for cement-retained dental implants and 0.89 mm (0.45–1.33 mm) for screw-retained dental implants.^11^ Moreover, peri-implant disease was associated with residual cement in patients with predisposition for periodontal disease, arguing for the use of screw-retained dental implants in susceptible patients.^12^

In our study, no correlation was found between the KM width and PIM/PBL. Similarly, Adibrad et al.^39^ evaluated sixty-six functioning dental implants supporting overdentures and observed that, although the mean bone loss was higher for implants with narrow zones of keratinized mucosa, the difference was not significant. Adell et al.^1^ also failed to find a correlation between implant survival or success rates and the presence of KM. On the other hand, Chung et al.^40^ have demonstrated increased levels of plaque and inflammation around implants in the absence of KM. Another study.^41^also evaluated the response of peri-implant tissue in the presence of KM in 276 implants placed in 100 patients. Although the GI, PI, and PD were not significantly different in patients with or without keratinized gingiva, these authors observed that mucosal recession and marginal bone resorption were significantly increased in the dental implants with deficient keratinized mucosa. Therefore, in general, these results suggest that the presence of an appropriate amount of keratinized gingiva is beneficial for long-term maintenance and management, as well as for areas requiring esthetics.^4^

The limitations of this cohort study include the small sample size and the short-term follow-up that may underestimate the impact of implant- and/or patient-related factors on peri-implant bone loss. The findings observed in this cohort study should be evaluated in further studies with larger samples and longer follow-up periods. Moreover, the self-report nature of the data, particularly in the evaluation of systemic disorders and smoking habits, combined with the lack of knowledge on the degree of systemic status control is also considered to be a study limitation.

## Conclusion

Within its limitations, this 1-year prospective cohort study found a PIM incidence of 44.8% and a mean of peri-implant bone loss of 0.35 ± 1.89 mm in the dental implants evaluated. No influence of implant- and patient-related factors on PIM and PBL could be observed, except for the type of implant connection. PIM incidence was significantly higher in implants with internal connection type after 1 year of follow-up.
